# Assessment of Day-to-Day Functioning in Prodromal and Early Huntington Disease

**DOI:** 10.1371/currents.RRN1262

**Published:** 2011-09-12

**Authors:** Anthony L Vaccarino, Terrence Sills, Karen E. Anderson, Jean Endicott, Joseph Giuliano, Mark Guttman, Aileen K Ho, Peter Kupchak, Jane S. Paulsen, John H. Warner, Janet Williams, Ken Evans

**Affiliations:** ^*^Research Methods, Ontario Cancer Biomarker Network, Toronto, Ontario, Canada; ^‡^Department of Psychiatry and Department of Neurology, University of Maryland, School of Medicine, Baltimore, MD USA; ^§^Department of Psychiatry, Columbia University College of Physicians and Surgeons, New York, New York; ^¶^CHDI Foundation, Inc.; ^#^Division of Neurology, Department of Medicine, University of Toronto, Toronto, Ontario Canada; ^**^School of Psychology and Clinical Language Sciences, University of Reading, U.K.; ^‡‡^Department of Psychiatry, The University of Iowa Carver College of Medicine, Iowa City, IA, USA; ^§§^Department of Biostatistics, CHDI Foundation, Princeton NJ USA and ^¶¶^College of Nursing, The University of Iowa, Iowa City, IA

## Abstract

The Functional Rating Scale Taskforce for pre-Huntington Disease (FuRST-pHD) is a multinational, multidisciplinary initiative with the goal of developing a data-driven, comprehensive, psychometrically sound, rating scale for assessing symptoms and functional ability in prodromal and early Huntington disease (HD) gene expansion carriers. The process involves input from numerous sources to identify relevant symptom domains, including HD individuals, caregivers, and experts from a variety of fields, as well as knowledge gained from the analysis of data from ongoing large-scale studies in HD using existing clinical scales. This is an iterative process in which an ongoing series of field tests in prodromal (prHD) and early HD individuals provides the team with data on which to make decisions regarding which questions should undergo further development or testing and which should be excluded. We report here the development and assessment of the first iteration of interview questions aimed to assess functional impact in day-to-day activities in prHD and early HD individuals.

## Introduction

Earliest clinical manifestations of Huntington disease (HD) are poorly characterized, and there is a need for clinical scales specifically designed to measure early changes in HD gene expansion carriers. The Functional Rating Scale Taskforce for pre-Huntington Disease (FuRST-pHD) is a multinational, multidisciplinary collaboration to develop a valid functional rating scale to assess changes in symptom severity in HD gene expansion carriers who do not yet meet criteria for a formal clinical diagnosis (prodromal HD or prHD) or are early manifest.[1] Indeed, although formal research diagnosis is currently based on motor impairment, longitudinal data have demonstrated that there are many changes that occur before the presence of motor signs that meet conventional diagnostic criteria for manifest disease, including motor and non-motor signs and symptoms.[2] [3] Furthermore, scales developed to assess signs and symptoms and used to make a formal diagnosis (i.e., UHDRS)[4] were developed in manifest gene expansion carriers and thus may not be sensitive to changes in prodromal HD.[5] [6] Such a measurement tool is essential to better understand the earliest manifestations of HD and to evaluate novel therapies early in the course of disease.

In assessing therapeutic benefit, improvement can occur after treatment with respect to signs and symptoms, functional ability, and quality of life. As outlined by the US Food and Drug Administration (FDA):

*"... evidence of improved symptoms alone will only support claims specific to improvement of the symptoms and would not support a general claim related to improvement in a patient’s ability to function or the patient’s psychological state.”* [7]

Therefore, functional assessments, particularly in combination with symptomatic assessments, would support broader claims with respect to patient outcomes. 

Measures of functional impact assess the impact of disease on the ability of the patient to function normally; changes that should be meaningful/impactful to the patient. Two commonly used measures of function in people with prHD are Total Function Capacity (TFC)[4] and Functional Assessment Scale (FAS).[8] The TFC is a clinician rating scale with questions on occupation, finance, domestic chore, activities of daily living and level of care. The FAS contains more detailed questions on occupation, financies, activities of daily living, domestic chorse, level of care, and physical activities, with binary yes/no responses. Although each assesses important components of day-to-day functioning, in a multinational, multisite study of people with prHD, over 88% of participants scored at ceiling levels of the TFC and FAS.[6] Despite these limitations, occupational decline, and loss of ability to independently manage finances or to drive safely were most commonly noted among people who had not yet been diagnosed with HD.[9] The TRACK-HD study includes the SF36 which assesses physical and social interaction aspects of function.[10] Exploratory qualitative studies with people who have prHD document that functional changes can encompass work, as well as a wide range of daily activities, some of which require social interactions.[11] These findings support the need for measures that are sensitive to changes in daily function in people with prHD and those in the early HD stages. 

FuRST-pHD has established an inclusive process for scale development using input from numerous sources, including prHD and early HD individuals, family members, companions, and experts from a variety of fields, as well as from ongoing large-scale HD studies using existing clinical scales.[1] As part of the process, an inclusive series of “Working Groups” of individuals with clinical and/or scale development expertise were formed to review existing data and develop interview questions within the specific domain under study. Once these interview questions are developed, they are distributed to trained raters for beta testing in prHD and early HD individuals. This is an iterative process, in which changes or deletions (as appropriate) are made based on empirical evidence obtained during field testing; the modified questions are then tested during subsequent iterations so that the list can ultimately be winnowed to select optimal items for scale inclusion. 

We report here the development and assessment of the first iteration of interview questions aimed to assess day-to-day function in prHD and early HD individual. 

## Methods

Two-day Working Group meetings were held held in Princeton, NJ (May 4-5, 2010) and New York, NY (August 31-September 1, 2010). The working group charge was to review available evidence and develop interview questions to assess day-to-day functioning prHD and early HD.   

### Evidence Reviewed

Data Mining. 

As part of the process of developing items to assess defined symptom clusters, we have benefited from the use of data from ongoing studies investigating the symptomatology and progression of prHD and HD that are accessible to the FuRST pHD program, including PREDICT-HD and REGISTRY. These data provide provide rich and useful information about the expression of symptoms and day-to-day functioning in the target population, and differentiation of early changes from those expressed in advanced disease. Data assessing day-to-day functioning in prHD and HD were reviewed and considered by the working group in developing the interview questions, including:

- Unified Huntington's Disease Rating Scale-Total Functional Capacity (PREDICT-HD and REGISTRY)
- Functional Assessment Scale (PREDICT-HD)
- SF-36 Health-related Quality of Life (REGISTRY)

Patient and Companion Input. The FDA views input from participants, companions and family members as an essential element in developing valid clinical assessment tools.[7] To ensure that the scale reflects concepts that are important from the participant's perspective, patient/companion focus groups were held to identify early symptoms experienced by HD gene carriers and to determine the functional impact of these symptoms. All focus group had IRB/EC approval and all participants provided informed consent.

Face-to-face focus groups were held in a number of countries using the local languages (France, Netherlands, United Kingdom, United States, Portugal, and Spain) with all participants (prHD, early HD, and companions, n=101) being asked a series of open-ended questions related to symptom occurrence in prHD. A number of symptoms were reported as being bothersome and interfering with day-to-day activities, and assessed within symptoms domains (i.e., psychiatric, motor and cognitive).[12] [13] [14] [15]

To collect additional patient input about specific areas of day-to-day function, an exploratory self-report telephone interviews were conducted with 16 people with prHD or early HD to explore specific areas of day-to-day functioning, including household chores, paid work, ability to get around, shopping, eating and preparing meals, telephone use, hobbies, and social interactions.[11]

Expert Opinion and Experience of Participants. In addition to reviewing existing data, working group participant experiences and opinion were also discussed, both within HD and in other movement-related disorders, including Parkinson's disease. 

### Development of Interview Questions 

After review of existing data, the following relevant daily functioning domains were identified:

- Work
- Household
- Relationships
- Driving
- Finances
- Mobility
- Other (i.e., telephone use, eating and swallowing)

Interview questions were developed to probe the impact in the prHD and early HD population. The FuRST-pHD has adopted a semi-structured clinician-administered interview similar to that used for the GRID-HAMD. The GRID format directs the rater to score symptom frequency and intensity separately, while giving them clear scoring anchors, a semi-structured interview guide, and overall definitions. This method has been employed successfully and is user-friendly, with acceptable agreement among independent raters.[16] The working group developed interview questions, including structured interview guides, scoring conventions, scoring anchors, and symptom definitions. Following the meeting, draft interview questions were circulated for comment on a shared internet site (Sharepoint).

Based on a review of the evidence, 23 interview questions were developed for field testing (Table 1).

**Table 1.** Interview Questions

**Table:** 

**Interview Question**	**Description/Definition**
**Work-Impatience**	Degree to which impatience impacts relations with others at primary place of work
**Work-Productivity**	Ability to perform their expected tasks in their primary workplace
**Work-Self assessment ^a^**	Self-assessment of his/her productivity at primary work activities
**Work-Comments ** **^a^**	Comments on problems with individual’s productivity in the workplace
**Work-Quality ** **^b^**	Quality of work product
**Work-Initiation**	Degree to which individuals need to push themselves to initiate tasks at the workplace
**Work-Completion**	Degree to which individuals need to push themselves to complete tasks at the workplace
**Household -Productivity**	Ability to perform their expected tasks in household chores
**Household -Self-assessment ** **^a^**	Self-assessment of his/her productivity in performing household chores
**Household -Comments ** **^a^**	Comments on problems with individual’s productivity in performing household chores
**Household -Quality ** **^b^**	Quality of performance of household chores
**Household –Initiation**	Degree to which individuals need to push themselves to initiate chores and tasks at home
**Household –Completion**	Degree to which individuals need to push themselves to complete chores and tasks at home
**Family Relations ** **^b^**	Ability to maintain his/her most important familial relationship(s)
**Non-Familial Relations ** **^b^**	Ability to maintain his/her non-familial relationships
**Driving-Limitations ** **^b^**	Degree to which the individual limits their driving
**Driving-Confidence**	Degree to which the individual is confident about their driving
**Driving-Comments ** **^a^**	Comments from others on problems with individual’s driving abilities
**Driving-Negative emotions**	Emotional responses to situations or people while driving
**Finances ** **^b^**	Ability to manage his/her finances
**Mobility**	Ability to get around and be mobile/independent
**Eating/swallowing ** **^a^**	Ability to eat and drink to maintain their usual dietary intake and nutritional status
**Answering phone/door ** **^a^**	Problems experienced in answering the door and phone

^a^

Assessed as frequency only; ^b^Assessed as intensity only 

### Field Testing of Interview Questions

Field testing of interview questions in prHD (UHDRS Diagnostic Confidence Level < 4) and early HD (within 5 years from onset of clinical motor signs) was carried out at independently contracted sites. All data collection sites had IRB/EC approval, and all participants provided informed consent. Prior to conducting the clinical interview, all raters were trained (via webinar or in person) to ensure that all trainees had an adequate conceptual understanding for administering and scoring each of the items. Interview questions were tested in two separate batches of 8 questions (Work and Mobility) and 15 questions (Household, Relationships, Driving, Eating, Answering phone/door). To foster an environment of open discussion, interviews were conducted without participants' family members or companions present.

### Data Analysis 

The distribution of the composite score for each individual item was compiled, and summary statistics associated with each item score were computed. Distributions of item scores for HD and prHD subgroups were statistically compared using the non-parametric Mann-Whitney U test. 

Non-parametric item response analyses were performed to determine the relationship between scores on the individual interview questions and total score. Item Response Theory has been demonstrated to be useful in evaluating the performance of individual items (symptoms) on rating scales, by assessing the relationship between a score assigned to an item and the overall severity of the disease.[17] [3] IRT software (TESTGRAF) was used to generate Option Characteristic Curves (OCCs) that display the probability of a particular option score (i.e., a score of 0, 1, 2, 3, 4) on each Interview Question as a function of overall level of severity. In the present analyses, total motor item score was used as a measure of severity. To illustrate this, Figure 1 depicts a hypothetically ‘‘ideal’’ item from an item response perspective, which is characterized by a clear identification of the range of severity scores over which an option is most likely to be endorsed, rapid changes in the curves that correspond to changes in severity, and an orderly relationship between the weight assigned to the option and the region of severity over which an item is likely to be endorsed. As such, OCCs provide a graphical representation of how informative a particular item (or symptom) is as a measure of illness. Frequency distribution of option scoring within each interview question were also generated.

**Figure:**
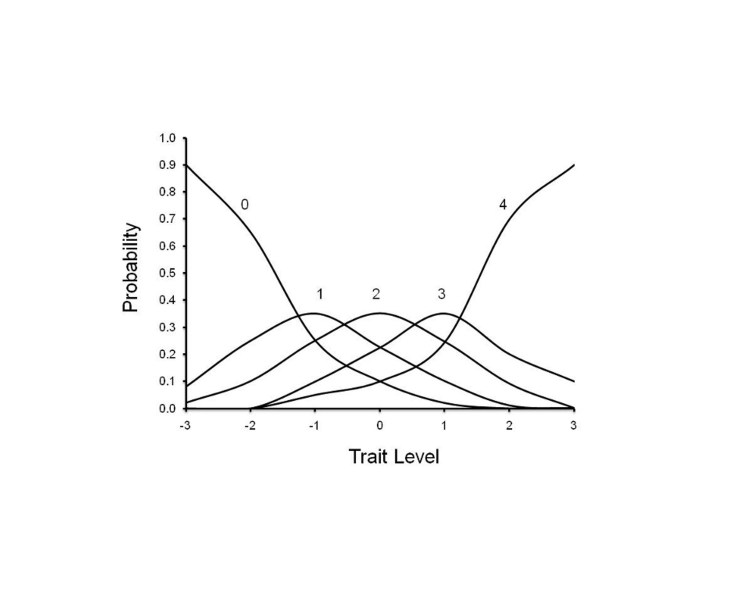
**Figure 1**. OCC for a hypothetically ‘‘ideal’’ item. The OCC shows the probability (*y*-axis) of endorsing a particular option for the item at different levels of the trait (*x*-axis).

Interview questions which were found to produce scoring and discrimination across ranges of overall severity were selected for further testing. For the subsets of well-endorsed items from each of the two batches of interview questions, total subscale scores for prHD and HD subjects were computed and compared statistically using the Mann-Whitney U test. The measure of internal consistency of each subscale was estimated using Cronbach's alpha, and the corrected item-total correlation (between each individual item score and the total of the other selected items in the subscale) was computed. Correlations between the total subscale score and scores of individual questions from the same batch that were not selected for further testing were also computed.  

## Results

A total of 192 CRFs were completed (8-item CRF, N=102; 15-item CRF, N=90). The participant demographic characteristics are shown in Table 2.

**Table 2.** Demographic Characteristics

**Table:** 

** **	**All Subjects**	** prHD**	** HD**
**Sample size**	N=192	N=112 (58%)	N=78 (41%)
**Male gender**	N=85 (44%)	N=37 (33%)	N=47 (60%)
**Age** **(mean,range)**	47.3 (18-79)	43.7 (18-71)	52.5 (23-79)

Follow-up Working Group meetings were held (via webinar) to review data and make recommendations in moving forward, including item deletion and modification/refinement. The FDA PRO Guidance was used to guide the decision making process.[7]  

OCCs and scoring frequency distributions were generated for each of the interview questions. Of the 23 tested, 11 interview questions were found to produce scoring and discrimination across ranges of overall severity:

*✓  Work-Impatience ✓  Work-Productivity ✓  Work-Self-Assessment ✓  Work-Initiation ✓  Work-Completion ✓  Household-Productivity ✓  Household-Self-Assessment ✓  Household-Initiation ✓  Household-Completion ✓  Eating/Swallowing ✓  Mobility*

With respect to the six well-endorsed items from the first batch of interview questions, Cronbach's alpha was 0.86 with respect to the entire subset of subjects, 0.91 with respect to the prHD subgroup and 0.81 with respect to the HD subgroup. Among these six items, all corrected item-total correlations were 0.50 or higher with respect to the HD subgroup (with the exception of that for the Mobility item), and all corrected item-total correlations were 0.58 or higher with respect to the prHD subgroup (Table 3). The mean total composite score with respect to the six well-endorsed items from the first batch of interview questions was 3.16 in prHD subjects and 4.36 in HD subjects; the difference in mean scores was not statistically significant (*p* = 0.10, Mann-Whitney U test). The only item from the first batch of interview questions for which the mean score was significantly higher in HD than in prHD subjects was the Mobility item (*p* = 0.005, Mann-Whitney U test). 

With respect to the five well-endorsed items from the second batch of interview questions, Cronbach's alpha was 0.83 with respect to the entire subset of subjects, 0.82 with respect to the prHD subgroup and 0.84 with respect to the HD subgroup. Among these five items, all corrected item-total correlations were 0.55 or higher with respect to the HD subgroup (with the exception of that for the Eating/Swallowing item), and all corrected item-total correlations were 0.61 or higher with respect to the prHD subgroup (with the exception of that for the Eating/Swallowing item) (Table 4). The mean total composite score with respect to the five well-endorsed items from the second batch of interview questions was 3.41 in prHD subjects and 4.41 in HD subjects; the difference in mean scores approached statistical significance (*p* = 0.08, Mann-Whitney U test). The two items from the second batch of interview questions for which the the mean score was significantly higher in HD than in prHD subjects were the Eating/Swallowing item (*p* < 0.001, Mann-Whitney U test) and the Household - Completion item (*p* = 0.03, Mann-Whitney U test).  

**Table 3.** ******Correlations between Interview Question Scores (Batch I)

**Table:** 

**Item **	**Item-total correlation (all subjects)**	**Item-total correlation (prHD subjects)**	**Item-total correlation ** **(HD subjects)**
Work -Impatience	0.545	0.576	0.497
Work - Productivity	0.730	0.832	0.624
Work - Self-assessment	0.811	0.868	0.769
Work -Comments	0.593	0.580	0.596
Work -Quality	0.592	0.595	0.583
Work -Initiation	0.654	0.756	0.583
Work -Completion	0.670	0.763	0.643
Mobility	0.555	0.737	0.391

****Table 4. Correlations between Interview Question Scores (Batch II)

**Table:** 

**Item**	**Item-total correlation (all subjects)**	**Item-total correlation (prHD subjects)**	**Item-total correlation (HD subjects)**
Household- Productivity	0.738	0.611	0.834
Household- Self-Assessment	0.665	0.795	0.548
Household - Comments	0.612	0.549	0.623
** **Household - Quality	0.641	0.585	0.659
** **Household - Initiation	0.691	0.644	0.788
** **Household - Completion	0.751	0.767	0.730
Family Relations	0.226	0.152	0.330
Non-Familial Relations	0.291	0.285	0.266
Driving - Limitations	0.562	0.722	0.434
Driving - Confidence	0.660	0.676	0.622
** **Driving - Comments	0.270	0.206	0.244
** **Driving – Negative Emotions	0.273	0.319	0.212
** **Finances	0.446	0.542	0.419
Eating and Swallowing	0.330	0.275	0.313
** **Answering phone or door	0.365	0.447	0.261

It was agreed that these 11 interview questions would be modified accordingly and tested in subsequent iterations; examination of the OCCs provided data on which modifications should be made to improve item performance, including changes in wording and scoring options. For example, Figure 2 shows that for "Initiation of Work-related Tasks," the options with the highest probability of being scored for symptom intensity increased from "0" to "2" (moderate: Can begin tasks but with some difficulty, feel that you had to exert mental effort to get going); however, scores of 3 (severe: Can initiate tasks but only with prompting or cueing) and 4 (Can initiate tasks only with significant external persuasion) were never endorsed as the option with the highest probability, suggesting that assessment of severity is not captured based on comments/prompts received from others. With respect to symptom frequency, these data showed that when problems with initiation of tasks at the workplace do occur they were "much of the time/almost all of the time" and tended not to occur "occasionally" (see Figure 2). 

**Figure:**
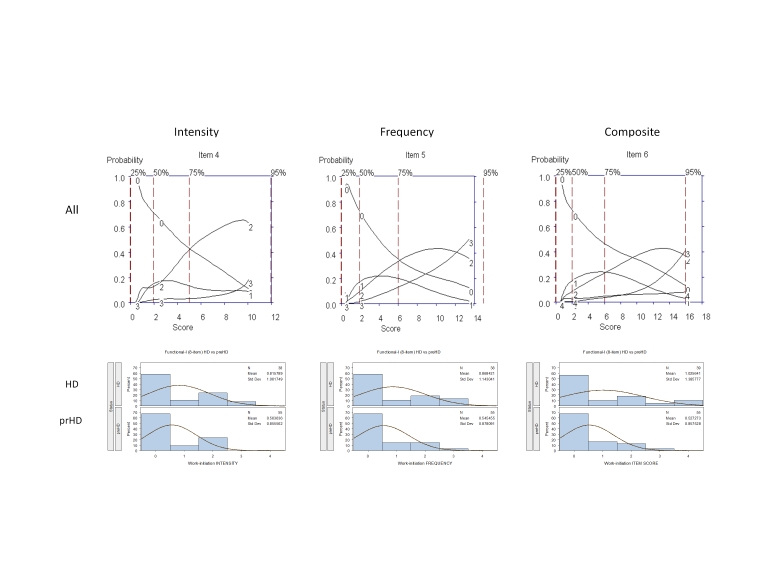
**Figure 2.** OCCs (All participants) and Frequency distribution (HD and prHD) for interview question assessing "Initiation of Tasks at the Workplace." Data are presented separately for symptom intensity (left row), frequency (middle row) and composite (right row) scores.

The remaining 12 questions were very rarely endorsed and a score of zero had the highest probability of being scored across most of the range of severity (Figure 3, as example): 

*✗  Work-Comments*

*✗  Work-Quality*

*✗  Household-Quality*

*✗  Household-Comments*

*✗  Family Relations*

*✗  Non-Family Relations*

*✗  Driving-Limitations*

*✗  Driving-Confidence*

*✗  Driving-Comments*

*✗  Driving-Negative emotion*

*✗  Finances*

*✗  Answering phone/door*

In general, the low frequency of response and poor discriminative properties limit the usefulness of these interview questions for assessment in prHD and early HD. Mean scores were significantly higher in HD subjects than in prHD subjects for four of the above 12 items (Household-Quality, Driving-Limitations, Driving-Confidence and Driving-Comments); none-the-less, these four items were all poorly endorsed in prHD and HD subjects. 

Figure 3 shows the OCCs and frequency distributions for three interview questions that assessed three areas related to driving: "Degree to which the individual limits their driving," "Degree to which the individual is confident about their driving," and "Emotional responses to situations or people while driving." For all of these areas of driving a score of zero had the highest probability of being endorsed across a broad range of severity. 

With respect to reporting of negative responses to driving, although scores of zero had the highest probability of being endorsed across the entire range of severity, options 1 (Mild emotional response but no overt expression) and 2 (Some outward expression; brief) were endorsed with some frequency in both prHD and HD (see Frequency distributions, Figure 3). Indeed, this finding is consistent with previous results for interview questions aimed to assess experiences of anger and irritability.[12] However, the correlation between the score for this item and the total composite score from the 5 highly-endorsed items in the second batch of interview questions was low (Table 4), and Cronbach’s alpha would have decreased upon addition of this item to the set of well-endorsed interview questions.


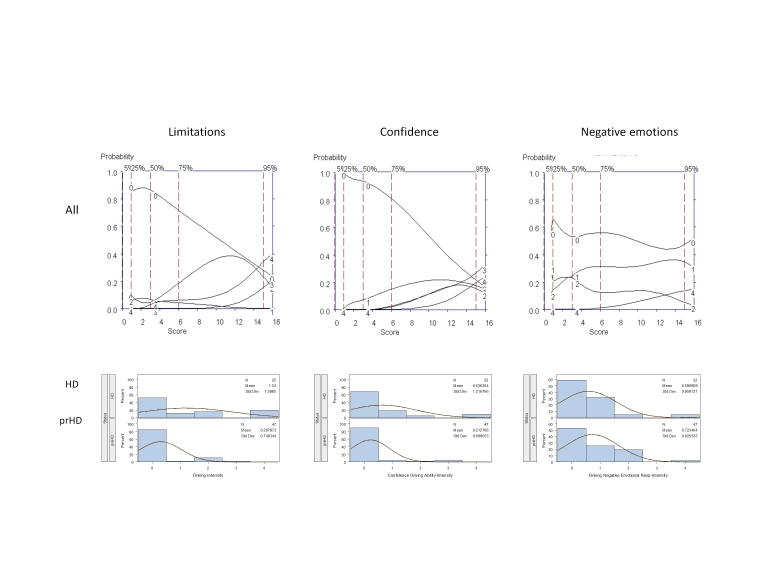


**Figure 3.** OCCs (All participants) and Frequency distribution (HD and prHD) for interview questions assessing Diving: "Limitation" (left row), "Confidence" (middle row) and "Negative Emotions" (right row).

It was agreed that these 12 interview questions should be removed from subsequent iterations on the basis of Relevance (Reported as not relevant by a large segment of the population of interest) and Response Range (A high percentage of patients respond at the floor) as outlined in Table 1 of the FDA PRO Guidance.[7]

## Discussion

As part of FuRST-pHD, interview questions are being developed to assess functional ability in prodromal and early HD gene expansion carriers. We report here the development and beta testing of first iteration interview questions designed to assess changes in day-to-day functioning. 

Of interest was that higher scores were reported in HD than prHD, including assessments of mobility, eating/swallowing and completion of household chores. These results suggest that these changes are sensitive to disease progression in HD, and may reflect the presence of motor- and/or cognitive-related deficits that can interfere with the ability to perform these tasks. Indeed, *ability to detect change *and *differences* *among groups hypothesized a priori to be different (i.e., known groups validity) *are critical measurement properties that will need to be considered for validation and use of the final instrument in clinical trials.[7]

In the development of these interview questions, participants were given the choice to indicate if the the activity was not actually engaged in the preceding two-week period; that is whether the interview question was "not-applicable." For example, with respect to driving, 10% of prHD and 32% of HD participants reported either "never driving" or "did not drive in the last two weeks." Similarly, 14% of prHD and 10% of HD participants reported that they did not do their finances. These cases were treated as 'missing data' and omitted from the analyses. It is important to recognize, however, that these cases do not represent 'missing data' in the usual sense, and are certainly not missing at random. Thus, the data may not represent an unbiased estimate of functional ability. It is also possible that family members or companions may contribute to the decreased activity within these domains because of the companion's recognition of difficulties, thus modifying participant's behavior. Although it is not possible to determine precisely the reasons for the lack of the behaviour, it is clear that the percentage participants that reported either "never driving" or "did not drive in the last two weeks" was higher in HD than prHD (32% vs 10%, respectively). This difference may reflect an increase in disability related to progression in HD. 

How to incorporate 'missing data' for functional items not applicable to all participants in a composite scale score will need to be addressed by the FuRST-pHD. The 'missing data' clearly cannot be coded as a score of '0' as this represents lack of disability or low symptom burden in the current scale. Neither is casewise deletion a suitable option. It may be that the scale will be divided into sub-scales, and then the average score of each sub-scale combined to derived a composite index. It is also possible to impute the 'missing' value, and there are various methods for doing this.[18] The decision on how to handle the cases with 'missing data' will need to be taken prior to finalizing the scale for validation.

Further to the reduced applicability of the driving items to the prHD and particularly early HD group, the low frequency of scoring of these items may reflect a general lack of insight or impaired self-awareness (i.e., anosognosia),[19] [20] or under-reporting due potential implications over licensing. Other factors such as the complexity of the driving task and potential compensation by patients or other drivers around them for any behavioural changes make this less amenable to self-report. Nevertheless, driving changes did arise in the more selective focus group where individuals may have been more willing and open to discussing negative aspects of driving,[11] and there was indeed more signal for the HD group relative to prHD on three of the four items here. In this respect driving remains a potentially important aspect of function but may be better examined through direct assessment of actual or simulated performance of the driving task, or possibly proxy report, rather than self-report. 

Based on the results, eleven interview questions have been selected for further testing, have been modified accordingly by the working group, and are currently undergoing a second iteration of field testing. The results of the second iteration will be reported once completed. 

### Acknowledgments 

CHDI Foundation, Inc. – a not-for-profit research organisation whose mission is to rapidly and collaboratively discover and develop therapies that slow the progression of Huntington’s disease – initiated and sponsored the development of the FuRST-pHD. We thank Jamie Levey for her help coordinating the European focus groups, LaVonne Goodman for her help coordinating the USA focus groups, and Stacie M Vik and Barbara McQuaid for administrative assistance.

### Funding Information

FuRST-pHD is funded by CHDI. PREDICT-HD is supported by the National Institutes for Health, National Institute of Neurological Disorders and Stroke (NS40068) and CHDI Foundation, Inc

### Competing Interests

The authors have declared that no competing interests exist.

### FuRST-pHD Core Team

K Anderson, B Borowsky, K Evans, J Giuliano, M. Guttman. A Ho, JS Paulsen, T Sills, A Vaccarino

### Working Group

FuRST-pHD Core Team, D Craufurd, J Endicott, M Groves, J Williams

### Statistics

S Gilbert-Evans, P Kupchak, T Sills, A Vaccarino 

### Contributing Field Testing Sites and Coordinators

Birmingham and Solihull Mental Health, Birmingham, UK (Hugh Rickards, MD, Jenny Crooks, BA, Jan Wright, BA); Center for Movement Disorders, Markham, Ontario, Canada (Mark Guttman, MD, Irita Karmalkar, BA, Alanna Sheinberg, BA, and Adam Singer, BA); University of Melbourne, AU (David Ames, MD, Edmond Chiu, MD, Phyllis Chua, MD, Olga Yastrubetskaya, PhD, Joy Preston, Anita Goh, D.Psych, and Angela Komiti, BS, MA); University of Iowa, Iowa City, IA, USA (Leigh Beglinger, PhD, Thomas Wassink, MD, Patricia Ryan, MSW, MA, Stephen Cross, BA, Mycah Kimble, BA, Stacie Vik, BA); Huntington Disease Drug Works, Seattle, WA, USA (LaVonne Goodman, MD); North York General Hospital, Toronto. Ontario, Canada (Clare Gibbons, MS, Jeanne Kennedy, BScNEd, RN, and Wendy Meschino, MD)

### Focus Groups

Portugal (J Ferreira, T Mestre), Spain (A Martínez Descals), France (A Durr, C Jauffret), The Netherlands (R Bos, R Roos, M-N Witjes-Ané), UK (R Fullam, O Handley, J Naji); HD Drug Works, Seattle, USA (L Goodman)

### Corresponding Author

Anthony L Vaccarino, avaccarino@ocbn.ca
